# Interdisciplinary Collaborations in Digital Health Research: Mixed Methods Case Study

**DOI:** 10.2196/36579

**Published:** 2022-05-04

**Authors:** Grit Krause-Jüttler, Jürgen Weitz, Ulrich Bork

**Affiliations:** 1 Department of Visceral, Thoracic and Vascular Surgery Faculty of Medicine, Technische Universität Dresden University Hospital Carl Gustav Carus Dresden Germany; 2 National Center for Tumor Diseases Dresden (NCT/UCC) German Cancer Research Center (DKFZ), Heidelberg Helmholtz-Zentrum Dresden - Rossendorf (HZDR) Dresden Germany

**Keywords:** team science, interdisciplinary, research collaboration, digital health, team processes

## Abstract

**Background:**

Digital innovations in medicine are disruptive technologies that can change the way diagnostic procedures and treatments are delivered. Such innovations are typically designed in teams with different disciplinary backgrounds. This paper concentrates on 2 interdisciplinary research teams with 20 members from the medicine and engineering sciences working jointly on digital health solutions.

**Objective:**

The aim of this paper was to identify factors on the individual, team, and organizational levels that influence the implementation of interdisciplinary research projects elaborating on digital applications for medicine and, based on the results, to draw conclusions for the proactive design of the interdisciplinary research process to make these projects successful.

**Methods:**

To achieve this aim, 2 interdisciplinary research teams were observed, and a small case study (response rate: 15/20, 75%) was conducted using a web-based questionnaire containing both closed and open self-report questions. The Spearman rank correlation coefficient was calculated to analyze the quantitative data. The answers to the open-ended questions were subjected to qualitative content analysis.

**Results:**

With regard to the interdisciplinary research projects investigated, the influencing factors of the three levels presented (individual, team, and organization) have proven to be relevant for interdisciplinary research cooperation.

**Conclusions:**

With regard to recommendations for the future design of interdisciplinary cooperation, management aspects are addressed, that is, the installation of a coordinator, systematic definition of goals, required resources, and necessary efforts on the part of the involved interdisciplinary research partners. As only small groups were investigated, further research in this field is necessary to derive more general recommendations for interdisciplinary research teams.

**Trial Registration:**

German Clinical Trials Register, DRKS00023909, https://www.drks.de/drks_web/navigate.do?navigationId=trial.HTML&TRIAL_ID=DRKS00023909 ; German Clinical Trials Register, DRKS00025077, https://www.drks.de/drks_web/navigate.do?navigationId=trial.HTML&TRIAL_ID=DRKS00025077

## Introduction

### Background

Digital innovations in medicine are disruptive technologies that can change the way diagnostic procedures and treatments are delivered. Innovation is usually designed in teams with different disciplinary backgrounds. Collaboration between professionals and experts from different educational backgrounds can release creative energies [1]. However, for successful interdisciplinary cooperation, certain basic principles must be observed. At present, projects require the cooperation of people with different knowledge and skills, who together consider complex problems from interdisciplinary perspectives and pursue new paths. Interdisciplinary cooperation is particularly necessary for the development of new technical achievements such as health care technologies. However, a lack of knowledge about the skills of people from other disciplines and different *languages* and *cultures* leads to problems in the process of interdisciplinary research collaboration.

In contrast to projects where people from different disciplines work in different areas, that is, have different individual goals, people in interdisciplinary teams have goals that they can accomplish only if they work together. Therefore, interdisciplinarity refers to the mutual dependence of disciplines. To achieve common goals, procedures or methods must be *negotiated* between disciplines. If this can be achieved without conflict, existing methods can be improved or new activities can be created. It is important that team members are aware that goals are to be achieved together, which the individual disciplines cannot achieve. People from other disciplines must be granted skills, and *other opinions* must be taken seriously. All participants must be aware of their role in the team and organization, have respect for other disciplines, and see the common goal as their distinct goal.

However, this effort is worthwhile when innovative solutions for complex challenges arise by combining the strengths of all participants.

This paper concentrates on 2 interdisciplinary research projects involving 20 researchers working jointly at the interface of medicine and engineering sciences. Using sociological methods of qualitative and quantitative surveys, this study examined which factors influence the implementation of interdisciplinary research between medicine and engineering sciences and which approaches exist to successfully shape this form of collaborative research in the future.

The first interdisciplinary project considered is the ARAILIS (Augmented Reality and Artificial Intelligence Supported Laparoscopic Imagery in Surgery) project, which aims to develop a prototype for innovative computer-assisted surgery using augmented reality and artificial intelligence. It is designed to support surgeons in making decisions that increase accuracy and therefore reduce the likelihood of complications during liver surgery.

The second project is the interdisciplinary PROSPER (Platform for Operation Scheduling and Prediction Using Machine Learning) project, which aims to develop a platform that enables efficient and data-based decision-making for operating room (OR) planning processes through machine learning and the use of artificial intelligence. Using retrospective OR data and expert knowledge modeling, an automated solution is created that precisely predicts surgery duration, guarantees continuous planning adaptation, and enables day-based, flexible planning of all surgeries in multiple ORs for optimal resource use and OR efficiency.

In both projects, experts from medicine, computer, and further engineering sciences are researching interdisciplinarily.

The reasons for involving different scientific disciplines in solving medical research questions are multifaceted. The advantage of interdisciplinary collaboration is seen above all in the fact that a multidisciplinary approach to so-called *real-world problems* delivers more reliable results that are closer to application. In addition, especially when dealing with complex problems, such as digitalization in the health care system, additional expertise that is not available per se in the medical field is required [2,3].

In this study, both projects were jointly investigated, because they have comparable characteristics. In both projects, an interdisciplinary collaboration among engineers, computer scientists, and surgeons takes place, and they are working on a similar topic dealing with artificial intelligence to support the decision-making of surgeons. Therefore, the study team decided to investigate them together to obtain a larger data basis for describing and analyzing collaboration processes between these different disciplines. According to the previous project descriptions, differences can only be found in the concrete results that the projects are focusing on (surgery planning platform, image-based assistance system), but the way of collaboration; the different disciplines involved; and their way of cooperation are comparable in both considered projects.

### Brief Overview About the State of Research

Over the past 30 years, researchers have extensively dealt with the issues of teamwork and the cross-disciplinary composition of research teams. In this context, a definitional issue must first be addressed.

As Aboelela et al [4] pointed out on the basis of a literature review, previous research has found various forms of cross-disciplinary research collaboration. Their review explains that the forms described in the literature can be defined along a continuum in terms of the “quality of the actual integration of different disciplines,” the “degree of cooperation (interaction of the researchers involved, communication and exchange of information),” and regards the “outcome of the collaboration”; that is, a concretely achieved solution [4]. On the basis of their research results, the authors distinguish the concepts of “multidisciplinarity,” “interdisciplinarity,” and “transdisciplinarity” with respect to the characteristics “participants/discipline,” “problem definition,” “research style,” and “presentation of findings” [4].

In accordance with this review, for the purpose of this paper, interdisciplinary research is understood as follows:

any study or group of studies undertaken by scholars from two or more distinct scientific disciplines. The research is based upon a conceptual model that links or integrates theoretical frameworks from those disciplines, uses study design and methodology that is not limited to any one ﬁeld, and requires the use of perspectives and skills of the involved disciplines through-out multiple phases of the research process

4

So, in contrast to *multidisciplinarity*, it is not a matter of additive cooperation in which disciplines work on partial aspects and develop their own solutions in parallel, but rather of a mutual expansion and integration of methods and solution approaches and thus a mutual compensation of existing gaps in the respective discipline with regard to the problem to be solved [5,6].

Previous research has dealt with the topic of research work in cross-disciplinary teams under the umbrella term *team science*. Klein [7] provides a brief overview of its various strands and distinguishes the following three main research clusters: *international network of interdisciplinary research* [8], the *transdisciplinary team science* (TTS) [9-11], and the *transdisciplinary trans-sector, problem-oriented research with stakeholders in society* localized in Europe [12]. Although the work of the first and third research clusters does not focus on any research area per se, *TTS* focuses on the field of interdisciplinary medical research and its cooperation with other, nonmedical scientific disciplines with the aim of answering complex questions, such as the management of cancer or the digitalization of health care. Therefore, the (TTS) available research results proved to be particularly relevant to the results presented in this paper. For completeness, reference should be made to the field of *Interprofessional Health Practice and Education* [13], which focuses on interprofessional cooperation in medical care.

Generally, both TTS and Interprofessional Health Practice and Education deal with the questions of how social factors influence interdisciplinary collaboration and how collaboration must be organized to work successfully in interdisciplinary teams [11] in the aforementioned sense [4]. Compared with disciplinary research, interdisciplinary collaboration poses some challenges. To make individuals from different disciplinary backgrounds and with different organizational affiliations collaborate successfully, an increased effort for communication and a high investment of time are required, especially in the early stages of collaboration. These investments are necessary to develop a common understanding of the research question, to make the different objectives of the participants involved in the research project transparent, and to establish an understanding of the respective conditions in the participating organizations [14]. Therefore, interdisciplinarity was not a success. Numerous influencing factors at various levels promote the success of interdisciplinary research projects. Interdisciplinarity can only cause a real benefit if these influencing factors are known and considered when coordinating cooperation [15-17].

Publications available to date have identified the relevant factors that influence the success of interdisciplinary cooperation in the *individuals* involved, in the interaction within the *team*, and in the conditions for interdisciplinary research within the involved *organizations* [11,13,18].

However, successful interdisciplinarity has often been mentioned, but how can it be defined and measured? Tigges et al [14] provide an initial overview. There are 2 forms of measuring the success of interdisciplinary research work: first, the quantitative counting of results, such as publications or the amount of acquired external funds. Second, the most common type is the use of preformulated items for self-report to determine the individual perception of the involved researchers regarding the results of interdisciplinary research. Various instruments for the subjective assessment of the research process and the quality of interdisciplinary collaboration already exist, but have not yet been standardized. Such instruments can be found in various publications [18-24].

### Research Questions

The introduced ARAILIS and PROSPER projects can only achieve the planned research and development objectives if they implement successful cooperation between representatives from the disciplines of medicine, computer, and engineering sciences. On the basis of the briefly outlined state of research regarding interdisciplinary research cooperation in the medical context, this paper focuses on the *first question*, which relation exists between the individual attitudes of the researchers involved, their perceptions of the cooperative research process at the team level, organizational framework conditions, and functioning interdisciplinary research cooperation.

As a *second question*, this paper focuses on recommendations for shaping interdisciplinary research collaborations between medicine and engineering sciences. The goal is to draw conclusions from the results for the proactive design of the interdisciplinary research process and thus ensure the achievement of projects’ defined technical objectives with special attention to the maybe specific situation of interdisciplinary research in the medical context.

## Methods

### Overview

**Figure 1:**
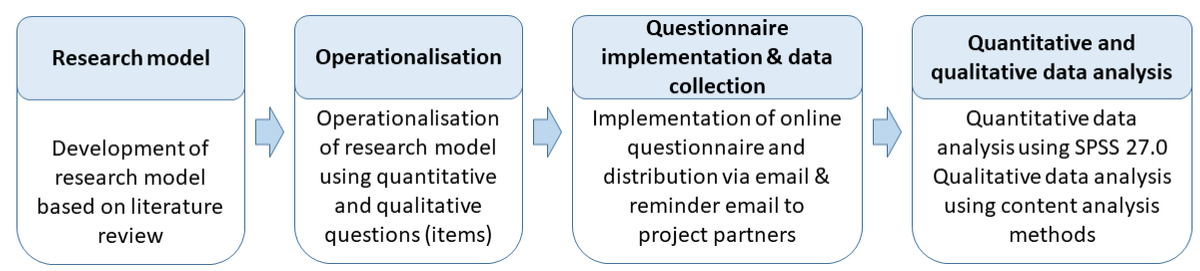
Research process of this study.

To answer these research questions, we conducted a small case study to investigate the introduced interdisciplinary projects. The study took place through a web-based questionnaire using the Lime Survey tool containing both closed and open questions for self-reporting. This study was conducted according to the process described in Figure 1.

**Figure 2:**
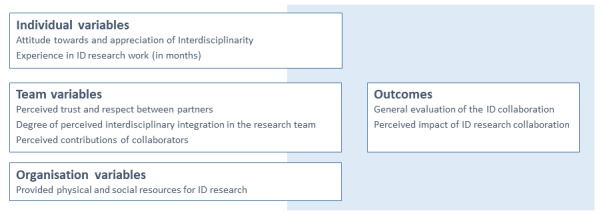
Research model for investigating variables that may be associated with a functioning interdisciplinary (ID) research collaboration.

For quantitative questions, we mainly used existing measurement methods [18-24]. The operationalization for measuring the different variables displayed in Figure 2, including their respective literature sources, is presented in Multimedia Appendix 1 [20,23]. With the use of open questions in the web-based questionnaire, the respondents were asked to formulate with their own words their current impressions about the implementation and design of interdisciplinary research work in the observed projects.

### Ethics Approval

No ethics committee approval was obtained for this anonymous survey trial in accordance with our internal review board guidelines (IRB00001473).

### Recruitment

In these 2 interdisciplinary projects, 20 researchers from different disciplines were involved. The participants were requested to complete the questionnaire. The survey was conducted in November and December 2020, when both projects were in the first year of their planned 3-year duration, meaning that some of the teams had not been working together for a long period. This investigation was planned as the first measurement point of a longitudinal study with at least one additional measurement point to observe the development of social interaction processes of project members over time in relation to the final project results. The links providing access to the web-based questionnaire were sent via email to define the final date. Two weeks after the first email invitation, all potential survey participants received a reminder. Finally, 15 (in some cases 16) responded to the survey. Studies based on data collected in web-based surveys from individuals normally reach response rates of approximately 53% (SD 20.4%) [25]. Therefore, in this study, it is assumed that a response rate of 75% allows reliable conclusions to be drawn for the project teams under investigation.

For quantitative data analysis, the Spearman rank correlation coefficient (SRCC) was calculated using SPSS (version 27.0; IBM Corporation). The SRCC is a nonparametric measure that can be applied to very small sample sizes [26]. The correlation coefficient was calculated at the item level. The results are presented in Tables 1-3, which show selected descriptive measures (mean and SD) as well as the calculation results for significant correlations of items that were used to operationalize the individual, team, and organizational variables and the defined outcomes listed in Figure 2. Owing to the rather small sample size, only very close relationships between the variables examined proved to be significant [26]. All items were rated by 15 of the 20 project members contacted. Items assessed by 16 of the 20 are marked in Tables 1-3.

The answers to the open questions were subjected to qualitative content analysis based on the approach provided by Mayring [27] to abstract and summarize their essential content.

**Table 1:** Mean, SD, Spearman rank correlation coefficient (SRCC; 1-sided hypothesis test), and *P* values for individual variables and outcomes.

				Value, mean (SD)		Productivity of collaboration meetings		Overall productivity of collaboration		Integration of results succeeds well		Development of common language succeeds well		Development of a common theoretical basis succeeds well		Our project team is successful		My subproject is successful		In general, collaboration has improved my research productivity
**Outcomes**																				
	**Productivity of collaboration meetings (n=16)**			3.13 (0.72)		—^a^		—		—		—		—		—		—		—
		SRCC																		
		*P* value																		
	**Overall productivity of collaboration (n=16)**			3 (81) (0 66)				—		—		—		—		—		—		—
		SRCC			0.30															
		*P* value			.13															
**In our project team**																				
	**Integration of results succeeds well**			3.67 (0.82)						—		—		—		—		—		—
		SRCC			−0.01		0.47													
		*P* value			.48		.04													
	**Development of common language succeeds well**			3.73 (0.59)								—		—		—		—		—
		SRCC			0.28		0.48		0.67											
		*P* value			.16		.03		.003											
	**Development of a common theoretical basis succeeds well**			3.60 (0.74)										—		—		—		—
		SRCC			−0.06		0.16		0.51		0.38									
		*P* value			.41		.28		.03		.08									
**Measured by results so far**																				
	**Our project team is successful**			3.67 (0.82)												—		—		—
		SRCC			0.05		0.61		0.66		0.39		0.30							
		*P* value			.43		.008		.003		.07		.14							
	**My subproject is successful**			3.73 (0.80)														—		—
		SRCC			0.06		0.48		0.60		0.29		0.20		0.93					
		*P* value			.41		.04		.01		.15		.23		<.001					
	**In general, collaboration has improved my research productivity**			4.07 (0.70)																—
		SRCC			−0.06		−0.02		0.43		0.38		0.74		0.20		0.20			
		*P* value			.42		.47		.05		.08		.001		.24		.24			
**Individual variables**																				
	**I am optimistic that ID^b^ research among project collaborators will lead to valuable scientific outcomes that would not have occurred without that kind of collaboration^c^ (n=16)**			4.63 (0.50)																
		SRCC			−0.17		0.35		0.20		0.07		0.26		0.51		0.40		−0.12	
		*P* value			.26		.09		.23		.40		.17		.03		.07		.33	
	**Participating in an ID team improves the results that are developed^c^**			4.20 (0.86)																
		SRCC			−0.16		0.23		0.37		0.06		−0.00		0.32		0.44		−0.13	
		*P* value			.28		.21		.09		.42		.50		.13		.05		.33	

^a^Not applicable.

^b^ID: interdisciplinary.

^c^What do you think about the interdisciplinary research process in the project from your individual point of view? Please rate your views using gradations 1 “strongly disagree,” 2 “somewhat disagree,” 3 “not sure,” 4 “somewhat agree,” and 5 “strongly agree.”

**Table 2:** Mean, SD, Spearman rank correlation coefficient (SRCC; 1-sided hypothesis test), and *P* values for team variables and outcomes.

					Value, mean (SD)			Productivity of collaboration meetings			Overall Productivity of collaboration			Integration of results succeeds well			Development of common language succeeds well			Development of a common theoretical basis succeeds well			Our project team is successful			My subproject is successful			In general, collaboration has improved my research productivity
**Outcomes**																													
	**Productivity of collaboration meetings (n=16)**			3.13 (0.72)			—^a^			—			—			—			—			—			—			—	
		SRCC																											
		*P* value																											
	**Overall Productivity of collaboration (n=16)**			3.81 (0.66)						—			—			—			—			—			—			—	
		SRCC				0.30																							
		*P* value				.13																							
**In our project team**																													
	**Integration of results succeeds well**			3.67 (0.82)									—			—			—			—			—			—	
		SRCC				−0.01			0.47																				
		*P* value				.48			.04																				
	**Development of common language succeeds well**			3.73 (0.59)												—			—			—			—			—	
		SRCC				0.28			0.48			0.67																	
		*P* value				.16			.03			.003																	
	**Development of a common theoretical basis succeeds well**			3.60 (0.74)															—			—			—			—	
		SRCC				−0.06			0.16			0.51			0.38														
		*P* value				.41			.28			.03			.08														
**Measured by results so far**																													
	**Our project team is successful**			3.67 (0.82)																		—			—			—	
		SRCC				0.05			0.61			0.66			0.39			0.30											
		*P* value				.43			.008			.003			.07			.14											
	**My subproject is successful**			3.73 (0.80)																					—			—	
		SRCC				0.06			0.48			0.60			0.29			0.20			0.93								
		*P* value				.41			.04			.01			.15			.23			<.001								
	**In general, collaboration has improved my research productivity**			4.07 (0.70)																								—	
		SRCC				−0.06			−0.02			0.43			0.38			0.74			0.20			0.20					
		*P* value				.42			.42			.05			.08			.001			.24			.24					
**Team variables**																													
	**Acceptance of new ideas^b^ (n=16)**			4.19 (0.91)																									
		SRCC				0.50			0.43			0.28			0.39			0.57			0.49			0.38			0.21		
		*P* value				.02			.048			.15			.07			.01			.03			.08			.23		
	**Communication among collaborators^b^ (n=16)**			3.88 (0.62)																									
		SRCC				0.55			0.42			0.24			0.15			0.11			0.41			0.27			0.00		
		*P* value				.01			.06			.20			.30			.35			.06			.16			.50		
	**Resolution of conflicts among collaborators^b^ (n=16)**			3.94 (0.57)																									
		SRCC				0.22			0.18			−0.01			0.32			0.44			0.25			0.10			0.17		
		*P* value				.21			.25			.48			.13			.05			.19			.36			.27		
	**Ability to accommodate different working styles of collaborators^b^ (n=16)**			4.13 (0.81)																									
		SRCC				0.52			0.53			0.30			0.52			0.20			0.52			0.37			0.05		
		*P* value				.02			.02			.14			.02			.24			.02			.09			.43		
	**Integration of research methods from different fields^b^ (n=16)**			3.69 (0.70)																									
		SRCC				−0.02			0.38			0.67			0.23			0.40			0.67			0.51			0.19		
		*P* value				.47			.08			.003			.21			.07			.003			.03			.25		
	**Integration of theories and models from different fields^b^(n=16)**			3.63 (0.72)																									
		SRCC				−0.01			0.34			0.76			0.37			0.50			0.59			0.43			0.19		
		*P* value				.48			.10			.001			.08			.03			.01			.05			.25		
	**Involvement of collaborators from diverse disciplines^b^ (n=16)**			4.19 (0.66)																									
		SRCC				−0.23			0.47			0.55			0.48			0.51			0.62			0.46			0.12		
		*P* value				.20			.03			.02			.04			.03			.007			.04			.34		
	**High motivation for collaboration^c^**			4.40 (0.51)																									
		SRCC				0.11			0.49			0.31			0.36			0.45			0.31			0.24			0.52		
		*P* value				.34			.03			.13			.09			.045			.13			.19			.02		
	**Reliable fulfillment of tasks taken over within the project team^c^**			3.93 (0.60)																									
		SRCC				0.40			0.71			0.51			0.38			0.11			0.43			0.29			0.17		
		*P* value				.0			.002			.03			.08			.35			.06			.15			.27		
	**Willingness to coordinate one’s own research work with the others in the project team and to work intensively with the other project members^c^**			4.27 (0.80)																									
		SRCC				−0.04			0.26			0.00			−0.04			0.49			0.27			0.13			0.58		
		*P* value				.44			.17			.50			.45			.03			.16			.32			.01		
	**Interest in other disciplines involved and willingness to recognize other disciplines as equivalent^c^**			4.33 (0.62)																									
		SRCC				−0.09			0.27			0.53			0.66			0.49			0.35			0.19			0.66		
		*P* value				.38			.16			.02			.004			.03			.10			.25			.004		

^a^Not applicable.

^b^When thinking about the researchers collaborating on the project, how do you evaluate the following aspects? Please use the gradation 1 “inadequate,” 2 “poor,” 3 “satisfactory,” 4 “good,” or 5 “excellent”!

^c^With regard to your experiences in the project so far, what impressions do you have regarding the research contributions of your collaborators? How do the following statements apply: Please evaluate the mentioned issues using gradation 1 “does not apply at all,” 2 “does more likely apply,” 3 “does partly apply,” 4 “does more likely apply,” or 5 “does strongly apply.”

**Table 3:** Mean, SD, Spearman rank correlation coefficient (SRCC; 1-sided hypothesis test), and *P* values for organization variables and outcomes.

					Value, mean (SD)			Productivity of collaboration meetings			Overall productivity of collaboration			Integration of results succeeds well			Development of common language succeeds well			Development of a common theoretical basis succeeds well			Our project team is successful			My subproject is successful			In general, collaboration has improved my research productivity
**Outcomes**																													
	**Productivity of collaboration meetings (n=16)**			3.13 (0.72)			—^a^			—			—			—			—			—			—			—	
		SRCC																											
		*P* value																											
	**Overall productivity of collaboration (n=16)**			3.81 (0.66)						—			—			—			—			—			—			—	
		SRCC				0.30																							
		*P* value				.13																							
**In our project team**																													
	**Integration of results succeeds well**			3.67 (0.82)									—			—			—			—			—			—	
		SRCC				−0.01			0.47																				
		*P* value				.48			.04																				
	**Development of common language succeeds well**			3.73 (0.59)												—			—			—			—			—	
		SRCC				0.28			0.48			0.67																	
		*P* value				.16			.03			.003																	
	**Development of a common theoretical basis succeeds well**			3.60 (0.74)															—			—			—			—	
		SRCC				−0.06			0.16			0.51			0.38														
		*P* value				.41			.28			.03			.08														
**Measured by results so far**																													
	**Our project team is successful**			3.67 (0.82)																		—			—			—	
		SRCC				0.05			0.61			0.66			0.39			0.30											
		*P* value				.43			.008			.003			.07			.14											
	**My subproject is successful**			3.73 (0.80)																					—			—	
		SRCC				0.06			0.48			0.60			0.29			0.20			0.93								
		*P* value				.41			.04			.01			.15			.23			<.001								
	**In general, collaboration has improved my research productivity**			4.07 (0.70)																								—	
		SRCC				−0.06			−0.02			0.43			0.38			0.74			0.20			0.20					
		*P* value				.42			.47			.05			.08			.001			.24			.24					
**Organization variables**																													
	**Physical resources for ID^b^ research: availability of physical space (eg, office, laboratory etc)^c^**			3.80 (0.94)																									
		SRCC				0.23			0.45			0.37			0.13			0.46			0.46			0.53			0.13		
		*P* value				.20			.045			.09			.32			.04			.04			.02			.32		
	**Physical resources for ID research: availability of electronic or other resources for collaboration between remote research sites (knowledge management systems, online platforms and cloud services, etc)^c^**			3.93 (1.22)																									
		SRCC				0.61			0.10			−0.06			−0.21			−0.21			−0.11			0.01			−0.29		
		*P* value				.008			.37			.42			.23			.23			.35			.49			.14		
	**Social resources for ID research: my involvement in an ID research project is highly appreciated by my supervisors^c^**			4.07 (0.59)																									
		SRCC				0.55			0.31			0.02			0.28			0.07			0.37			0.38			0.15		
		*P* value				.02			.13			.47			.16			.40			.09			.08			.29		
	**Social resources for ID research: my involvement in an ID research project is highly appreciated by my colleagues^c^**			3.73 (0.70)																									
		SRCC				0.33			0.54			0.57			0.55			0.31			0.74			0.64			0.32		
		*P* value				.11			.02			.01			.02			.13			.001			.005			.12		

^a^Not applicable.

^b^ID: interdisciplinary.

^c^Considering the provided institutional or social resources for conducting the interdisciplinary research work in the project, how do you evaluate the availability of the following issues? For your evaluation, please use the gradation 1 “inadequate,” 2 “poor,” 3 “satisfactory,” 4 “good,” or 5 “excellent”!

### Collection of Quantitative Data

In accordance with the state of research briefly presented earlier, the closed survey questions focused on the subjective assessment of the individual, team, and organization variables that might be associated with a functioning interdisciplinary research collaboration.

Figure 2 lists the variables collected. Multimedia Appendix 1 contains their concrete operationalization in the survey questionnaire based on existing instruments [19-22] and the development of their own measurements.

*Individual variables* measure the respondent’s personal attitude toward and evaluation of interdisciplinary research collaboration as well as the respondent’s own experience with it. It is assumed that a positive attitude and long-term interdisciplinary experience are associated with positive perceptions of interdisciplinary outcomes.

*Team variables* cover the aspects of perceived trust between partners, the self-assessed degree of interdisciplinary integration in the research team, and the extent to which partners contribute to team outcomes. It is assumed that the positive ratings of these variables are associated with the positive ratings of interdisciplinary outcomes.

*Organization variables* refer to the support the respondents perceive for interdisciplinary research within their home organizations; for example, whether the necessary physical resources and social support from supervisors and colleagues are available for this.

*Outcomes* were measured by means of a general individual assessment of the results achieved so far and the productivity of the interdisciplinary research team. The collection of objective key figures, such as the number of publications, did not yield any results at this early point in the projects and will be collected again at later measurement points.

### Collection of Qualitative Data

Furthermore, the questionnaire included open-ended questions. Respondents answered the following self-developed questions (the sample size is provided in brackets):

1. What are the advantages and the disadvantages of interdisciplinary research work? (n=14)
2. Which facilitators and barriers for interdisciplinary research work do you personally perceive? Please think about aspects on individual, team, and organizational levels (eg, professorship or institute). (n=11)
3. Based on your experiences in interdisciplinary research so far, do you perceive differences between the collaboration with physicians and the collaboration with other disciplines? If yes, which differences do you see? (n=14)
4. When you recap your experiences in interdisciplinary research so far, which recommendations would you give to the project team and beyond to make interdisciplinary research collaborations successful? (n=13)

## Results

A demographic description of the surveyed project member sample is not provided because of the small group investigated and the possibility of an individual identification of responders; no demographic data of the project members were collected.

### Quantitative Results of the Correlation Analyses

Table 1 shows few significant results for the individual variables. Respondents who are very strongly optimistic that interdisciplinary will lead to valuable research results that would not have been produced otherwise also strongly agree that the project is successful (SRCC=0.51; *P*=.03). Respondents who strongly believe that interdisciplinary will improve the research results produced also strongly believe that their own subproject is successful (SRCC=0.44; *P*=.05). A significant relationship between the length of personal experience with interdisciplinary research and outcomes is not found for the studied sample and, therefore, not mentioned in the correlation table.

Numerous significant relationships emerged with the defined outcomes for the *team variables* listed in Table 2. Strong correlations (SRCC≥0.50) were shown for respondents who rated the acceptance of new ideas in the team as high. These respondents simultaneously perceived the high productivity of project meetings (SRCC=0.50; *P*=.02) and successful development of a common theoretical base (SRCC=0.57; *P*=.01). The perception of excellent communication in the interdisciplinary team was strongly associated with the productivity of project meetings rated high (SRCC=0.55; *P*=.01).

Individuals who rated the interdisciplinary team as very good at reconciling the different work styles of the collaboration partners, rated the productivity of work meetings (SRCC=0.52; *P*=.02), the overall productivity of the collaboration (SRCC=0.53; *P*=.02), the development of a common language (SRCC=0.52; *P*=.02), and, as measured by previous results, the success of the project team (SRCC=0.52; *P*=.02) as very high. The finding of a very strong integration of research methods from different fields is very closely related to the perception of a very high integration of results (SRCC=0.67; *P*=.003), successful work on the level of the overall project (SRCC=0.67; *P*=.003), and the respective subproject (SRCC=0.51; *P*=.03). The perception of a very successful integration of theories and models from different research fields is again strongly correlated with a high assessment of a successful integration of results (SRCC=0.76; *P*=.001), the perception of successful development of a collaborative theoretical basis (SRCC=0.50; *P*=.03), and successful project implementation (SRCC=0.59; *P*=.001). Respondents who assessed the involvement of collaborative partners from different disciplines were very good, rated the integration of results (SRCC=0.55; *P*=.02), the development of a collaborative theoretical base (SRCC=0.51; *P*=.03) as very high, and the project team as very successful (SRCC=0.62; *P*=.007). The perception of high motivation for collaboration among team members was significantly positively correlated with the perception that collaboration increased one’s research productivity (SRCC=0.52; *P*=.02). The perception that tasks taken on in the interdisciplinary team are reliably completed by team colleagues is closely related to the positive assessment of collaboration productivity (SRCC=0.71; *P*=.002) and to a perceived very successful integration of results in the team (SRCC=0.51; *P*=.03). An existing willingness to coordinate one’s own research with team members and to collaborate intensively with members is strongly associated with the assessment that interdisciplinary collaboration also greatly improves one’s research productivity (SRCC=0.58; *P*=.01). The impression that team members show interest in the disciplines involved and are willing to perceive them as equals is strongly associated with a perceived successful integration of results (SRCC=0.53; *P*=.02) and is strongly associated with the development of a common language (SRCC=0.66; *P*=.004) and the assessment that collaboration has improved one’s own research productivity (SRCC=0.66; *P*=.004).

The results for the *organization variables* contained in Table 3 point to the high relevance of physical resources (eg, offices and laboratories) for interdisciplinary research. In this case, significant correlations were found between the positive evaluation of their availability and collaboration productivity (SRCC=0.45; *P*=.045), the development of a common theoretical basis (SRCC=0.46; *P*=.04), a project perceived as successful (SRCC=0.46; *P*=.04), and a subproject perceived as successful (SRCC=0.53; *P*=.02) in the context of interdisciplinary collaboration. A positive evaluation of the availability of electronic resources for location-independent collaboration (knowledge management systems, web-based platforms, etc) shows a strong correlation with the assessment of a high productivity of meetings (SRCC=0.61; *P*=.008). As a side note, it should be added that perceived low availability of these electronic resources for collaboration shows a negative relationship with several aspects of outcome integration and success evaluation, although these are not significant. The availability of social resources is also relevant for outcome evaluation. High perceived supervisor support for interdisciplinary collaboration was significantly related to the highly rated productivity of project meetings (SRCC=0.55; *P*=.02). However, social support from colleagues in interdisciplinary research collaboration appears to be of greater importance. Respondents who perceived high appreciation by colleagues for their own involvement in interdisciplinary research simultaneously rated the productivity of collaboration highly (SRCC=0.54; *P*=.02), perceived strong integration of results in the interdisciplinary team (SRCC=0.57; *P*=.01), were more likely to rate the development of a common language as successful (SRCC=0.55; *P*=.02), and perceived both the overall project (SRCC=0.74; *P*=.001) and their own subproject (SRCC=0.64; *P*=.005) as more successful.

### Qualitative Results of the Open Questions

Textbox 1 summarizes the answers to question 1 regarding the advantages and disadvantages of interdisciplinary research.

These advantages are mainly seen in gaining new knowledge and methods from other disciplines. The respondents saw disadvantages in terms of the necessity of providing more time.

The answers to *question 2* are summarized in Textbox 2. These are facilitating factors for interdisciplinary research work in the establishment of a functioning project management (project manager, coordination of tasks, creation of common goals) but also in individual factors, such as the willingness to engage with other disciplines and to accept them.

In response to *question 3* about differences in collaboration with physicians compared to other disciplines, 6 (43%) of the 14 respondents stated that they did not perceive any differences. The remaining respondents mainly reported that they perceived physicians to be heavily involved in clinical work and therefore had less time for joint research work. One of the interviewees described it in such a way that physicians are seen more as outside experts who are only contacted when necessary, whereas the remaining multidisciplinary research partners are perceived as equal team members. One respondent also mentioned the problem of high data protection requirements for the use of patient data and the associated hurdles as a difference in cooperation with physicians compared with other disciplines.

Finally, the respondents provided their *recommendations* for the successful design of interdisciplinary research collaborations, which are summarized in Textbox 3. They are predominantly emphasizing the need for systematic collaboration management.

**Summarized answers regarding advantages and disadvantages of interdisciplinary research work (open question 1).**

**Advantages of interdisciplinary research (in comparison to monodisciplinary research)**

- Development of new ideas
- Knowledge transfer between different disciplines
- Leverage of different (technical) expertise and perspectives
- Broadening of mind and knowledge
- Experience of new methods
- Critical assessment of own methods, tools by other disciplines
- Solution of more complex problems by developing overall advanced systems

**Disadvantages of interdisciplinary research (in comparison to monodisciplinary research)**

- More time is needed for:
  Providing and gaining information, explanation
  Finding a common language
  Building a mutual understanding

**Summarized answers regarding facilitators and barriers for interdisciplinary research work (open question 2).**

**Perceived facilitators for interdisciplinary research**

- Supportive, reliable team members
- Independent project manager
- Good management or coordination by project leader
- Input from senior scientists
- Same or common goals
- Willingness to get involved in other scientific disciplines
- Acceptance that interdisciplinary needs more time

**Perceived barriers for interdisciplinary research**

- Different technical but also native languages (eg, different meanings of same terms)
- Dependency on work of others for own results
- Geographic distance between partners
- Focus on monodisciplinary research results
- Missing support for administrative issues
- Data protection problem regards medical data

**Summarized answers regarding recommendations for successful interdisciplinary research work (open question 4).**

**Recommendations for successful interdisciplinary research collaboration**

- Concrete definition of project objectives, workload, and requirements
- Installation of a project coordinator
- Implementation of regular meetings with progress reports
- Flexibility for changes and adaptions in the workplan
- Clarification of roles and expected contributions for each single project member
- Establishment of a common language

## Discussion

### Principal Findings

With regard to the quantitative results for the interdisciplinary research projects discussed here, influencing factors of all 3 levels presented (individual, team, and organization) have proven to be relevant for a cooperation that is perceived as successful. Positive individual attitudes toward interdisciplinary research work are related to positive outcomes. Pre-existing experience with interdisciplinarity did not play a role in the sample investigated, although this has already been shown in other studies [28]. The results of the team variables indicate that well-functioning group processes, in the sense of mutual acceptance and real integration of theories, methods, and approaches, are reflected in the perceived results of interdisciplinary collaboration. Thus, the results of the analyzed organizational factors indicate the high relevance of the provision of physical and social resources for successful interdisciplinary research collaboration. The high relevance of electronic resources for remote collaboration may be due to COVID-19 pandemic–related social distancing measures.

The answers to the open, more qualitative-oriented questions describe the advantages of interdisciplinary research work in getting to know new ideas, methods, and knowledge and integrating them into their own work. They also identified an advantage in the fact that more complex problems are solved that require the inclusion of different professional perspectives. They observed disadvantages, particularly the fact that this form of cooperation is more time-consuming, as more explanations and the establishment of a common understanding are necessary. Regarding recommendations for the future design of interdisciplinary cooperation, management aspects are addressed here, ie, goals, required resources, and necessary efforts on the part of the involved interdisciplinary research partners should be clearly defined in advance. Among other things, establishing a project coordinator and holding regular meetings are recommended. Furthermore, social aspects, such as the definition of the roles of each individual participant in the entire team and the establishment of a common language; for example, clarified common terms, should also be considered.

### Limitations

First, this study provides interesting insights into the projects introduced at the beginning of the article, but, at the same time, these insights are mainly limited to both projects. It is possible to derive some general conclusions and recommendations that are also covered by the existing research literature and results [11,17], but they must be viewed with some caution.

A further limiting factor was the statistical calculations that were performed. For the correlation analyses, the SRCC was calculated, which is a nonparametric measure that can be applied to very small sample sizes. It must be considered that these correlations do not allow any statement regarding a causal effect but only give hints about which aspects could be associated.

Moreover, the results were based on the self-reports of the responding researchers. Although personal opinions and intentions are relevant for individual behavior, this limitation to only one data source prevents additional data validation (eg, comparison of self and external assessment). In this regard, aspects such as common method bias and common method variance are of interest because all variables are measured using the same instrument [29,30]. Another limitation is that the variables considered in the research model (Figure 2) were very selective. These were chosen based on literature research, but not all identified influencing factors that were part of the questionnaire study became part of the data analysis presented in this paper. This is because no significant relationship was confirmed. Furthermore, it must be assumed that there is content overlap between the different variables presented in the research model (Figure 2). Further development of these instruments is necessary.

A further fact for consideration is the limited generalizability due to the survey period, which was during the second lockdown in Germany, caused by measures implemented due to the COVID-19 pandemic. In this regard, the mode of cooperation was based on web-based tools (videoconferences and email), and personal encounters did not occur. This certainly influenced the response behavior.

### Implications and Recommendations

Although the results are based on a small sample, it is possible to derive more general recommendations for the design and implementation of interdisciplinary research collaborations in digital health projects.

When providing recommendations for the design of successful interdisciplinary research collaboration, the levels of teams and organization can be considered because they are accessible to the direct influence of leadership and management. At the level of the concrete *team* that is collaborating, a project management regime should be implemented regarding the following aspects:

- Definition of a reliable and binding project plan including responsibilities, meetings, roles of all team members, timeline, and deadlines.
- Installation of a person who is and feels responsible for monitoring and complying with the plan (eg, project coordinator).
- Elaboration of a common understanding of the contents and objectives of the project.
- Establishment of team spirit and mutual trust as a precondition for openness and exchange of knowledge between research partners.

In addition, it must not be forgotten that the members of the interdisciplinary team are also members of the organizations where they are employed. At this *organizational level*, some aspects must be assured for successful collaboration to take place. As the results show, the following issues must be considered:

- Creation of an organizational atmosphere that demonstrates appreciation for interdisciplinary research work and is well-aware that it takes more time and effort in comparison to monodisciplinary research.
- Provision of social support (eg, recognition, affiliation, and instrumental assistance) by supervisors and colleagues for interdisciplinary research efforts.
- Deployment of an appropriate technical infrastructure that enables interdisciplinary collaborations even about spatial distance.

Regarding clinician scientists and their special role perceived as somehow external, organizational modes should be found to give them the opportunity for more integration in the whole interdisciplinary research team, which in turn will contribute to common understanding and trust and, therefore, positive results of the cooperative science process.

In this organizational context, *individual characteristics* could also be considered as far as personnel selection procedures are concerned. When a vacant position in an interdisciplinary research team has to be filled, potential recruits should be considered or selected that have already experience with interdisciplinary research or who at least seem to be open for that kind of cooperation and have the empathy to engage with other disciplines.

In the future, training courses that qualify team members and leaders for interdisciplinary research cooperation could be envisioned to enable them to act under the special conditions of interdisciplinary research. In this regard, more research is necessary [17,31].

### Outlook

As mentioned at the beginning of this paper, interdisciplinary research, especially in the context of the digitalization of medicine and health care systems, has become increasingly important. To design and manage this kind of collaboration successfully, it is necessary to identify the *adjusting screws* at different levels in research organizations and beyond. Leaders, researchers, and students must be sensitized and trained for this type of cooperation. Constant research on these social respectively human factors influencing collaboration is essential, mainly regarding content-related aspects of training for interdisciplinary research [17,32]. However, from a methodological point of view, more sophisticated study designs for monitoring interdisciplinary research collaboration are necessary, especially regarding multivariate influences. In this regard, success indicators for interdisciplinary research should be extended beyond the dominance of bibliometrics [17]. In addition, for future research and bigger samples than that of this study, demographic data characterizing the actors involved should be collected to provide more information about the transferability of results to other fields of concern. Furthermore, the concrete disciplinary composition of the respective project teams must be considered because the differences in working styles and professional cultures of single disciplines may also impact collaboration.

To some extent, the present case study shows that individual and team perceptions of success can also be used. Besides this, already available and helpful results for managing interdisciplinary projects from social science disciplines should be integrated, reflecting topics such as *transformational leadership* and its impact on creativity [33] or team support roles [34].

## Appendix

Multimedia Appendix 1 Sources of used self-report items for collecting quantitative data.

## Abbreviations

ARAILIS: Augmented Reality and Artificial Intelligence Supported Laparoscopic Imagery in Surgery
OR: operating room
PROSPER: Platform for Operation Scheduling and Prediction Using Machine Learning
SRCC: Spearman rank correlation coefficient
TTS: transdisciplinary team science
