# Supplementary material for: Interdisciplinary Collaborations in Digital Health Research: Mixed Methods Case Study
Source: JMIR Hum Factors. 2022 May 4;9(2):e36579. doi: 10.2196/36579 (PMC9118027; doi:10.2196/36579)
Supplement: Multimedia Appendix 1 [file humanfactors_v9i2e36579_app1.docx]

Multimedia Appendix 1. Sources of used self-report items for collecting quantitative data.

**Individual variables**

Attitude towards/ appreciation of Interdisciplinarity (Masse et al. 2008)

- I would describe myself as someone who strongly values interdisciplinary collaboration.
- Interdisciplinary research interferes with my ability to maintain knowledge in my primary area.
- I tend to be more productive working on my own rather than working as a member of an interdisciplinary research team. (R)
- Interdisciplinary research stimulates me to change my thinking.
- Interdisciplinary research has improved how I conduct research.
- I am optimistic that interdisciplinary research among collaborators will lead to valuable scientific outcomes that would not have occurred without that kind of collaboration.
- Because of my involvement in interdisciplinary research, I have an increased understanding of what my own discipline brings to others.
- I am comfortable working in an interdisciplinary project team.

Experience in ID research work (in months) (self-developed question)

1. How long have you been working in interdisciplinary research collaborations? Please insert your answer in 'months'!

**Outcomes**

General evaluation of the ID collaboration (TTURC initiative 2002)

1. In our project team, the integration of results succeeds well.
2. In our project team, the development of a common language succeeds well.
3. In our project team the development of a common theoretical basis succeeds well.
4. Measured by the results so far, our project team is successful.
5. Measured by the results so far, my subproject within the whole project is successful.

Perceived impact of ID research collaboration (Masse et al. 2008)

1. Productivity of collaboration meetings.
2. Overall productivity of collaboration.
3. In general, collaboration has improved my research productivity.

**Organisation variables**

Provided physical and social resources for ID research (TTURC initiative 2002 & self-developed items)

1. Availability of physical resources (e.g. computer equipment, software, supplies, etc.)
2. Availability of physical space (e.g. office, lab etc.)
3. Availability of electronic or other resources for collaboration between remote research sites (Knowledge management systems, online platforms and cloud services etc.)
4. My involvement in an interdisciplinary research project is highly appreciated by my supervisors.
5. My involvement in an interdisciplinary research project is highly appreciated by my colleagues.

**Team variables**

Perceived trust and respect between partners (Masse et al. 2008)

1. In general, I feel that I can trust the colleagues with whom I collaborate.
2. In general, I respect my collaborators.

Degree of perceived interdisciplinary integration in the research team (Masse et al. 2008)

1. Acceptance of new ideas.
2. Communication among collaborators.
3. Ability to capitalize on the strengths of different researchers.
4. Resolution of conflicts among collaborators.
5. Ability to accomodate different workings styles of collaborators.
6. Integration of research methods from different fields.
7. Integration of theories and models from different fields.
8. Involvement of collaborators from diverse disciplines.

Perceived contributions of collaborators (TTURC initiative 2002)

1. High motivation for collaboration
2. Reliable fulfillment of tasks taken over within the project team
3. Willingness to coordinate one's own research work with the others in the project team and to work intensively with the other project team members
4. Interest in the other disciplines involved and willingness to recognize other disciplines as equivalent
